# Mechanical Property of Long Glass Fiber Reinforced Polypropylene Composite: From Material to Car Seat Frame and Bumper Beam

**DOI:** 10.3390/polym14091814

**Published:** 2022-04-29

**Authors:** Bing Du, Zhengxuan Li, Huimin Bai, Qian Li, Changqi Zheng, Jingwei Liu, Feng Qiu, Zhenhua Fan, Hanjie Hu, Liming Chen

**Affiliations:** 1 Chongqing Key Laboratory of Nano–Micro Composite Materials and Devices, School of Metallurgy and Materials Engineering, Chongqing University of Science and Technology, Chongqing 401331, China; 2017441025@cqust.edu.cn (Z.L.); 2018440663@cqust.edu.cn (H.B.); 2018441597@cqust.edu.cn (Q.L.); 2019440636@cqust.edu.cn (C.Z.); 2019004@cqust.edu.cn (J.L.); 2019042@cqust.edu.cn (F.Q.); 2 College of Aerospace Engineering, Chongqing University, Chongqing 400030, China; 3 Innovation Center, Chongqing Polycomp International Corp., Chongqing 401321, China; china_fzh@163.com; 4 School of Aeronautics, Chongqing Jiaotong University, Chongqing 400074, China; huhj@gatri.cn; 5 The Green Aerotechnics Research Institute of CQJTU, Chongqing 401120, China

**Keywords:** long fiber reinforced composite, mechanical performance, theoretical analysis, finite element analysis, automobile component

## Abstract

Long Fiber Reinforced Thermoplastic (LFT) is a lightweight, high-strength, and easy-to-recycle new vehicle composite material, and has good mechanical properties, heat resistance, and weather resistance, which has found increasing application in automobile industry. It is of importance to understand the relationship between micro phase, macro-mechanical properties and the structural performance of automobile components. This article evaluates the performance of LFT from the level of material to automobile components. The mechanical properties of LFT were numerically and theoretically predicted to provide instruction for the next material choice. Two typical structural components, namely, car seat frame and bumper beam, were selected to evaluate the performance of LGF/PP compared with other competing materials in terms of mechanical properties and cost. In the case of the same volume, the seat frame of 40% LECT/PP composite material is lighter and cheaper, which is conducive to energy saving and emission reduction. It was shown that the 40% LECT/PA66 car bumper beam had a higher energy absorption ratio, lighter weight, higher specific energy absorption, and advantageous material cost. LFT is a promising candidate for existing automobile components with its performance fulfilling the requirements.

## 1. Introduction

The automotive industry’s requirements for energy conservation and emission reduction continue to increase. It has been shown that the weight of cars has been reduced by 10%, fuel consumption has been reduced by 6% to 8%, and exhaust emission has been reduced by 5% to 6% [1]. Thermoplastic composites have the characteristics of good mechanical properties and recyclability. After filling the thermoplastic composite resin matrix with fibers, it can reduce the weight and improve the degree of freedom of design [2]. The injection molding process is conducive to the integration of complex shape components and low-cost manufacturing. The amount and application structure of LFT in the automotive industry are increasing year by year, where LG/PP [3], LG/PA66 [4], etc. are of the most representative materials. It is of importance to understand the relationship between micro phase, macro-mechanical properties and the structural performance of automobile components.

Matsuda et al. [5] investigated the effects of fiber distribution on the elastic-viscoplastic behavior of long, fiber-reinforced laminates and found that fiber mass fraction and fiber length are the main factors affecting their mechanical properties. Yang et al. [6] found that only fibers that meet a certain length requirement in fiber-reinforced composite materials can play a reinforcing role. Obaid et al. [7] proposed an analytical model to predict the stress relaxation behavior of glass-fiber reinforced polypropylene composites and compared it with experimental and numerical results.

Li et al. [8] found that long glass fiber composite materials have better mechanical properties than short glass fiber composite materials. Chen and Cheng [9] studied the effective elastic modulus of planar orientation distribution and the transversely isotropic distribution of fibers based on the Eshelby–Mori–Tanaka theory. The effect of non-oriented, chopped-fiber composites by changing the fiber aspect ratio, fiber orientation and fiber volume fraction were compared via theoretical calculations. Sang et al. [10] predicted the low-temperature durability of short carbon fiber-reinforced polyamide 6 composites. Correlations were further developed for PA6 and CF/PA6 composites with moisture uptake to quantify tensile strength. Cui et al. [11] systematically studied the effects of strain rates on the mechanical response of long glass fiber-reinforced polypropylene composites (LGFRPPs). Niu [12] established an RVE model for predicting the mechanical properties of short fiber composites through direct homogenization methods and periodic boundary condition theory. Ogierman and Kokot [13] performed a multiscale modeling finite element analysis of short fiber-reinforced composite materials based on injection molding process simulation and investigated the effect of fiber orientation on the mechanical properties of the structural component. Zhou et al. [14] experimentally investigated the length, length distribution, and orientation of the fibers of glass fiber-reinforced polypropylene (GF-PP) parts. Kim et al. [15] investigated the strain rate dependent mechanical behavior of GFPP under a high strain rate. The impact simulation of the bumper beam was also performed in LS-Dyna. Wang et al. [16] numerically studied the performance characteristics of the long fiber composite material on the impact strength analysis and lightweight design of the car seat frame according to its performance. It was found that adding long glass fiber can be more effective in improving the mechanical properties of GF-PP-foamed parts as compared to solid parts.

In order to investigate the potential for LFT to be a candidate material for automobile lightweight structure, this article evaluates the performance of LFT from the level of material to automobile components. First, the mechanical properties of LFT were numerically and theoretically predicted to provide instruction for the next material choice. Second, two typical structural components, namely car seat frame and bumper beam, were selected to evaluate the performance of LFT compared with other competing materials in terms of mechanical properties and cost.

## 2. Performance Analysis of LGF/PP Material

For the composite with arbitrary-oriented fibers in space, the effective modulus can be calculated as Equations (1)–(6), according to the spatial angle average equation and the Mori–Tanaka method [17].

(1) $$\bar{C}=\left(3\bar{K},2\bar{G}\right)$$

where

(2) $$\bar{K}=K_{m}+\frac{V_{f}}{3\Delta }\left[\frac{1}{2\tilde{G}}+\frac{V_{m}}{3}\left(P_{2222}+P_{2233}+2P_{1111}-4P_{1122}\right)\right]$$

(3) $$\bar{G}=G_{m}+\frac{V_{f}}{30\Delta }\left[\frac{1}{\tilde{K}}+V_{m}\left(P_{1111}+2P_{2222}+2P_{2233}+4P_{1212}\right)\right]+\frac{V_{f}}{5}\left[\frac{1}{1/\left(2\tilde{G}\right)+2V_{m}P_{2323}}+\frac{1}{1/\left(2\tilde{G}\right)+2V_{m}P_{1212}}\right]$$

(4) $$\Delta =\left[\frac{2}{9\tilde{K}}+\frac{1}{6\tilde{G}}+V_{m}\left(P_{2222}P_{2233}\right)\right]\left(\frac{1}{9\tilde{K}}+\frac{1}{3\tilde{G}}+V_{m}P_{1111}\right)-2{\left(\frac{1}{9\tilde{K}}-\frac{1}{6\tilde{G}}+V_{m}P_{1122}\right)}^{2}$$

(5) $$\{\begin{array}{c} \tilde{K}=K_{f}-K_{m}\\ \tilde{G}=G_{f}-G_{m} \end{array}$$

(6) $$\{\begin{array}{c} P_{1111}=0\\ P_{2222}=P_{3333}=\frac{5-8\nu_{m}}{16G_{m}\left(1-\nu_{m}\right)}\\ P_{1122}=P_{2211}=P_{1133}=P_{3311}=0\\ P_{2233}=P_{3322}=-\frac{1}{16G_{m}\left(1-\nu_{m}\right)}\\ P_{1212}=P_{1212}=\frac{1}{8G_{m}}\\ P_{1212}=P_{1212}=\frac{1}{8G_{m}} \end{array}$$

$\bar{C},\bar{K},\bar{G},V$ are effective modulus, bulk modulus, shear modulus, and volume fraction, respectively. The subscript m,f, respectively, represent polymer matrix and fiber reinforcement. $P_{1111},P_{2222},P_{3333},P_{1122},P_{2233},P_{1212},P_{2323}$ are the components of the long-fiber type ellipsoidal inclusion tensor *P*.

**Figure 1:**
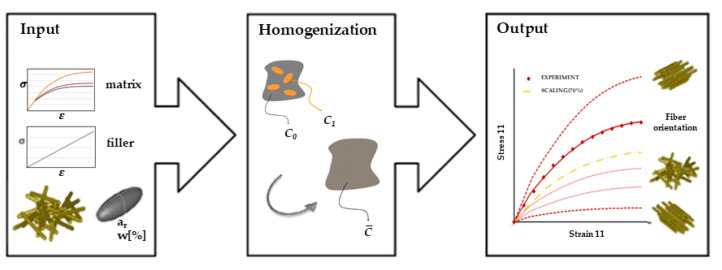
Flowchart of DIGIMAT-MF module [18].

The mechanical properties of LFT were predicted by micromechanical material modeling software DIGIMAT 2017 (MSC Software), specifically the DIGIMAT-MF module based on the average field homogenization method. The analysis process is shown in Figure 1. Reinforcement phase was set as ellipsoid with defined aspect ratio. Specific parameters of fiber and matrix, listed in Table 1, were imported into the software.

To verify the accuracy of prediction results of DIGIMAT on the macroscopic performance parameters of long-fiber composite materials, the prediction results of DIGIMAT were compared with theoretical calculations and experimental values. The component material performance parameters and microstructure parameters of LFT are shown in Table 1.

**Figure 2:**
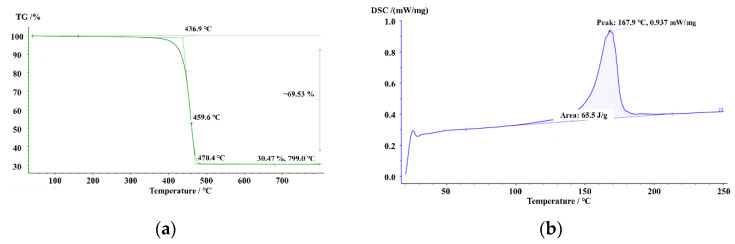
(**a**) TG curve; (**b**) DSC curve.

The injection-molded tensile specimens were supplied by Chongqing Polycomp International Corp. and their geometry was referred to ISO 527-2. A pellet with a length of about 12 mm was made from 30 mass% long ECT glass fiber and polypropylene. TG and DSC tests were conducted and results are listed in Figure 2. Injection molding machine (CJ80TB) was used to fabricate the dumbbell-shaped tensile specimen. The electronic universal testing machine (UTM5305SYXL) was utilized to assess the tensile properties of 30 mass% long ECT glass fiber-reinforced polypropylene (LECT/PP). The electronic extensometer (YYU-25) was used to measure the strain and the load was recorded by the testing machine. 

**Figure 3:**
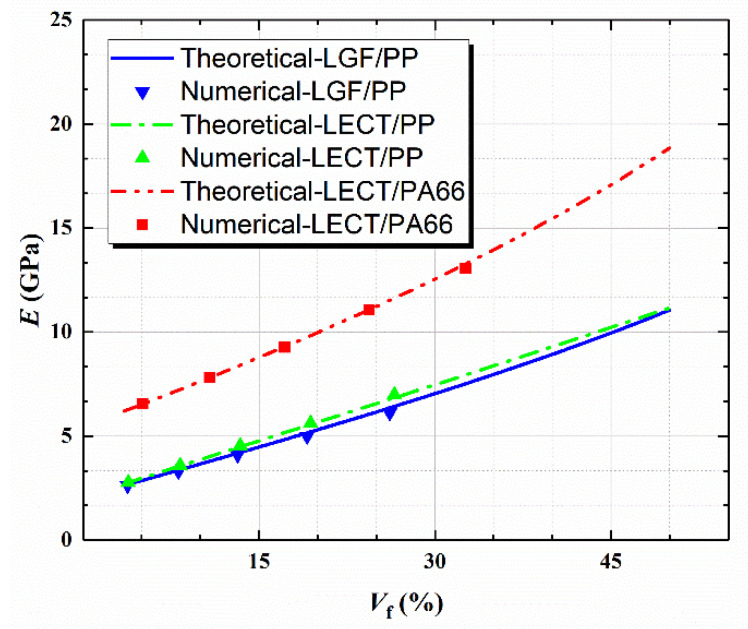
Theoretical and numerical tensile elastic modulus of LFT composite.

**Figure 4:**
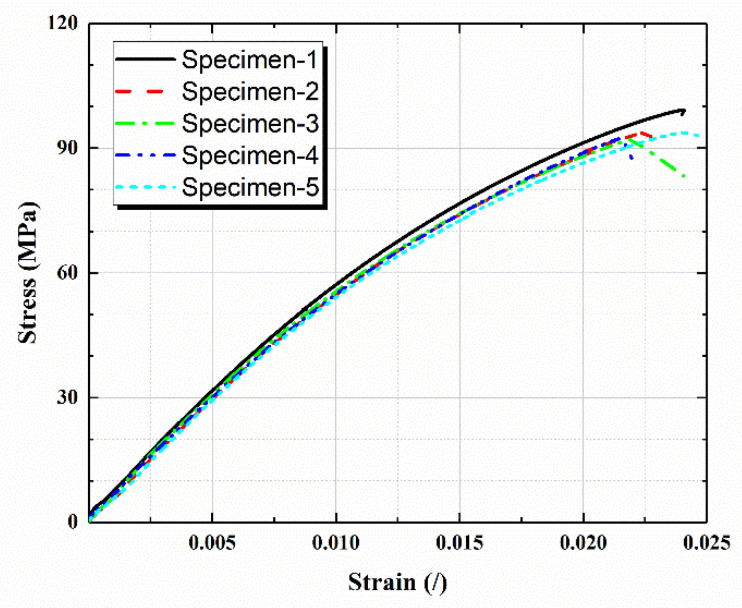
Tensile stress–strain curves of LGF/PP.

For the composite with arbitrary-oriented fibers in space, the effective modulus with different fiber contents was calculated as shown in Figure 3. The maximum error between the theoretical and numerical result was 3.75%, which proved that better consistency was achieved. According to the experimental result of LGF/PP, shown in Figure 4, the tensile elastic modulus and strength were obtained as and 6225 MPa and 92.4 MPa respectively. The numerical result from the DIGIMAT was 6390 MPa with a difference of 2.58%. In summary, the numerical results of DIGIMAT had good accuracy to meet the requirements of the following structural analysis.

## 3. Performance Analysis of LGF/PP Structure

Two typical structural components, namely, car seat frame and bumper beam, were selected to evaluate the performance of LGF/PP compared with other competing materials in terms of mechanical properties and cost.

### 3.1. Car Seat Frame

**Figure 5:**
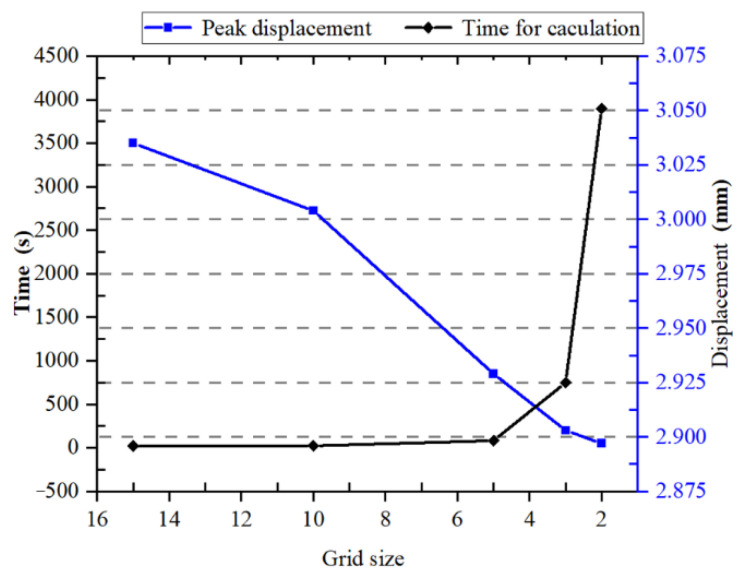
Mesh convergence test of different mesh sizes.

A typical car seat frame was selected and conditions of load and deformation were chosen according to GB 13057-2014, Strength of the Seats and Their Anchorage of Passenger Vehicles [19]. The geometric model was established and then imported into ABAQUS 6.14-1 (Dassault Systèmes). A load of 1300 N was exerted at 0.75 m above the end datum plane, and two bottom ends of the seat frame were fixed to constrain all degrees of movement. The geometry was meshed by a 4-node linear tetrahedral element (C3D4). A mesh convergence test with a different mesh size was firstly conducted, as shown in Figure 5. A mesh size of 3 mm was chosen for the next simulation considering the deformation and consuming time. Car seat frames with different materials, Q235A steel, AL 6061-T6, and 40% LECT/PP, were investigated, and the ideal elastoplastic constitutive relation was applied with the mechanical properties shown in Table 2. 

**Figure 6:**
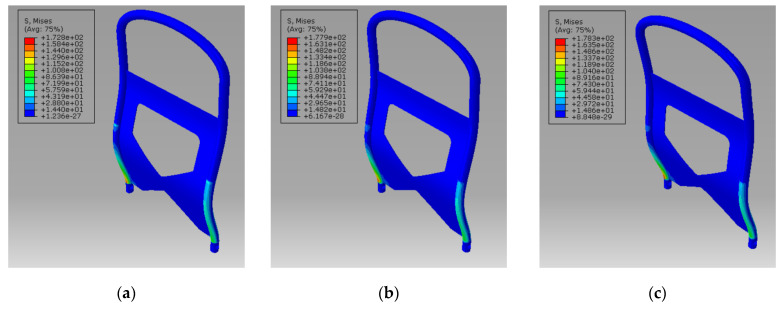
Stress contour of car seat frames: (**a**) Q235A; (**b**) AL 6061-T6; (**c**) 40% LECT/PP.

The stress contours of the car seat frames of different materials is shown in Figure 6. All maximum stresses were below the yield stress. Meanwhile, the maximum displacements at the highest points of the frames was obtained and are compared in Table 3. The displacement of all three seat frames met the requirement of GB 13057-2014. The displacement of 40% LECT/PP is the largest and that of Q235A steel is the smallest. Considering the difference in structural stiffness caused by the different elastic moduli of the materials, the product of the elastic modulus and the displacement of the highest point of the material was used as an index to evaluate the performance of the structure, and, thus, the product approaches a constant value. In the case of the same volume, the seat frame made from 40% LECT/PP composite material was found to be lighter and cheaper, which are qualities conducive to energy saving and emission reduction. It can be concluded that a seat back frame made of 40% LGF/PP composite material can achieve a lightweight structure under the premise that its performance meets the requirements.

### 3.2. Car Bumper Beam

**Figure 7:**
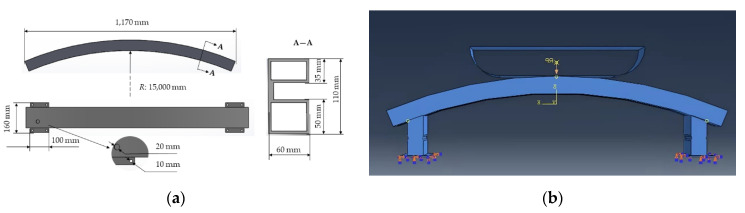
(**a**) Geometry of car bumper beam; (**b**) boundary condition of car bumper beam in ABABQUS.

The low velocity impact resistance performance of typical car bumper beams of different materials was also numerically investigated according to GB 17354-1998, Front and Rear Protective Devices for Passenger Cars [20]. The material parameters are listed in Table 2. The geometry was meshed by a 4-node linear tetrahedral element (C3D4) in HYPERMESH and then imported into ABAQUS. A weight of 1100 kg was attached in the rigid collider along with an initial impact velocity of 4 km/h. The collider was only allowed to move along the impact direction, and general contact was applied with the hard and frictionless contact property. The collider was tied with two energy absorption boxes whose ends were all fixed. The schematic diagram and boundary condition of a car bumper beam is shown in Figure 7.

**Figure 8:**
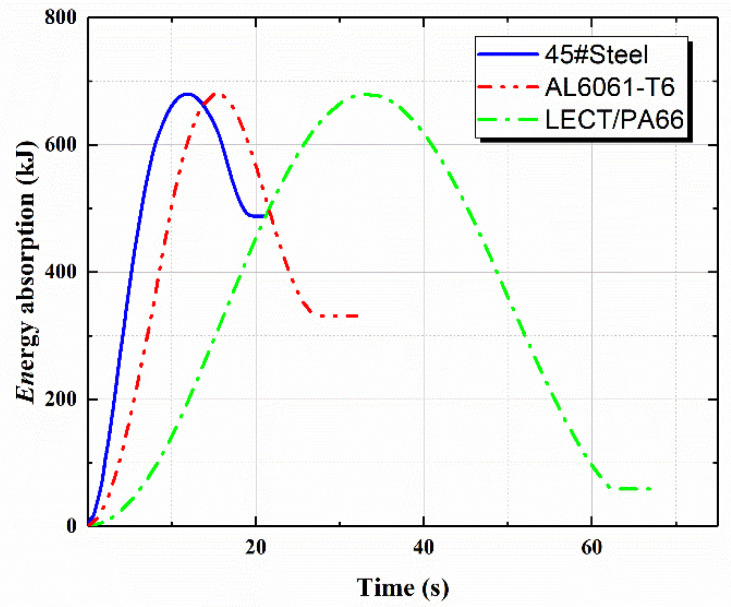
Comparison of energy absorption curves of competing materials.

The energy absorption–time curves are depicted in Figure 8. There were three stages, namely, impact contact, reaching the maximum amount of intrusion, and rebounding during the impact process. When the maximum amount of intrusion was reached, the velocity of the collider was zero. All kinetic energy was transferred into the elastic deformation energy and plastic deformation energy. Then, elastic deformation energy was relaxed and the collider was rebounded. The energy absorption ratio and amount of intrusion were taken as index to evaluate the performance of the car bumper beam. Energy absorption ratio was defined as the initial kinetic energy divided by plastic deformation energy as per Equation (7). The maximum amount of intrusion should be lower than 50 mm according to [12]. Energy absorption ratio and maximum amount of intrusion for three car bumper beams are shown in Table 4. The requirement in terms of maximum amount of intrusion was satisfied. It was shown that the 40% LECT/PA66 car bumper beam had a higher energy absorption ratio, lighter weight, higher specific energy absorption, and advantageous material cost.

(7) $$\eta =\frac{E_{\mathrm{Plastic}}}{E_{\mathrm{total}}}$$

## 4. Conclusions

This article evaluates the performance of LFT from the level of material to automobile components. The mechanical properties of LFT were numerically and theoretically predicted to give instruction for the next material choice. Two typical structural components, namely, car seat frame and bumper beam, were selected to evaluate the performance of LGF/PP compared with other competing materials in terms of mechanical properties and cost. LFT is a promising candidate for existing automobile components, with the performance fulfilling all requirements. The specific conclusions are as follows:

(1) The maximum error between the numerically predicted elastic modulus of the LFT and the theoretical calculation value is 3.74%, indicating that the performance results of the long fiber composite material predicated by the DIGIMAT software conform to the theoretical calculation model. The numerically predicted elastic modulus result for 6390 MPa is in good accordance with experimental results of 6225 MPa. The proposed numerical and theoretical methods had good enough accuracy to meet the requirements of the following structural analysis.
(2) Compared to Q235A steel and AL 6061-T6, in the case of the same volume, the seat frame composed of 40% LECT/PP composite material is lighter and cheaper, which is conducive to energy saving and emission reduction. It can be concluded that a seat back frame made of 40% LGF/PP composite material can achieve a lightweight structure under the premise that its performance meets the requirements.
(3) The energy absorption ratio and maximum amount of intrusion of three car bumper beams, namely 45# steel, AL 6061-T6, and 40% LECT/PA66, are 35.3%, 56.8%, and 96.0%, respectively. The requirement for the maximum amount of intrusion was satisfied. It was shown that the 40% LECT/PA66 car bumper beam had a higher energy absorption ratio, lighter weight, higher specific energy absorption, and advantageous material cost.

In future research, the effect of injection molding process parameters on the performance of structural components will be conducted utilizing the co-simulation of mold flow analysis software and ABAQUS to evaluate these findings more comprehensively.

## Figures and Tables

**Table 1:** Material parameters of used resins and fibers.

Performance Parameter	PP	PA66	E-Glass	ECT
Density ρ, g/cm^3^	0.91	1.22	2.52	2.57
Elastic modulus E, MPa	2070	5450	90,000	72,000
Poisson’s ratio (ν)	0.42	0.288	0.2	0.2
Yield strength ($\sigma_{s}$, MPa)	33.1	115	2465	2465
Fiber length (*l*, mm)	12			
Fiber diameter (*d*, μm)	14			
Critical fiber length (*l*_0_, mm)	3.1			

**Table 2:** Mechanical properties of competing materials.

Material	*E*/MPa	ν	$\sigma_{s}/\mathrm{MPa}$
Q235A	212,000	0.288	235
45#steel	209,000	0.269	355
AL 6061-T6	69,000	0.33	275
40% LECT/PP ^1^	8247.1	0.33	187
40%LECT/PA66 ^1^	14,123	0.33	221

^1^ Predicted by DIGIMAT. ν and $\sigma_{s}$ are Poisson’s ratio and Yield strength, respectively.

**Table 3:** Comparison between car seat frames made of different materials.

Material	Displacement U/mm	*E* × *U* /MPa·mm	Density/ g/cm^3^	Weight/kg	Unite Cost CNY/kg	Total Cost /CNY
Q235A	3.035	6.43 × 10^5^	7.86	8.454	4.8	40.58
AL 6061-T6	9.328	6.37 × 10^5^	2.7	2.904	38	110.35
40% LECT/PP	79.572	6.56 × 10^5^	1.3	1.398	23.8	30.76

**Table 4:** Comparison between car bumper beams made of different materials.

Material	Density/ g/cm^3^	Displacement *U*/mm	Energy Absorption Rate *η*/%	Weight/kg	Unite Cost CNY/kg	Total Cost /CNY
45#steel	7.89	7.26	35.32	13.785	6	82.71
6061-T6Al alloy	2.70	11.20	56.76	4.717	38	179.26
40%LECT/PA66	1.54	23.25	95.92	2.691	32.8	88.25

## Data Availability

The data presented in this study are available on request from the corresponding author.
